# An Artificial Reaction Promoter Modulates Mitochondrial Functions via Chemically Promoting Protein Acetylation

**DOI:** 10.1038/srep29224

**Published:** 2016-07-04

**Authors:** Yutaka Shindo, Hirokazu Komatsu, Kohji Hotta, Katsuhiko Ariga, Kotaro Oka

**Affiliations:** 1 Department of Biosciences and Informatics, Faculty of Science and Technology, Keio University, 3-14-1 Hiyoshi, Kohoku-ku, Yokohama, Kanagawa 223-8522, Japan; 2 MANA, National Institute for Materials Science, 1-1 Namiki, Tsukuba, Ibaraki 305-0044, Japan

## Abstract

Acetylation, which modulates protein function, is an important process in intracellular signalling. In mitochondria, protein acetylation regulates a number of enzymatic activities and, therefore, modulates mitochondrial functions. Our previous report showed that tributylphosphine (PBu_3_), an artificial reaction promoter that promotes acetylransfer reactions *in vitro*, also promotes the reaction between acetyl-CoA and an exogenously introduced fluorescent probe in mitochondria. In this study, we demonstrate that PBu_3_ induces the acetylation of mitochondrial proteins and a decrease in acetyl-CoA concentration in PBu_3_-treated HeLa cells. This indicates that PBu_3_ can promote the acetyltransfer reaction between acetyl-CoA and mitochondrial proteins in living cells. PBu_3_-induced acetylation gradually reduced mitochondrial ATP concentrations in HeLa cells without changing the cytoplasmic ATP concentration, suggesting that PBu_3_ mainly affects mitochondrial functions. In addition, pyruvate, which is converted into acetyl-CoA in mitochondria and transiently increases ATP concentrations in the absence of PBu_3_, elicited a further decrease in mitochondrial ATP concentrations in the presence of PBu_3_. Moreover, the application and removal of PBu_3_ reversibly alternated mitochondrial fragmentation and elongation. These results indicate that PBu_3_ enhances acetyltransfer reactions in mitochondria and modulates mitochondrial functions in living cells.

Mitochondria are essential organelles for cellular energy metabolism and intracellular signalling for cell death, and protein acetylation plays important roles in the modulation of mitochondrial functions^1^,^2^,^3^. Acetylation levels of mitochondrial proteins are regulated by the NAD^+^-dependent deacetylase activity of sirtuins. The mammalian genome encodes seven sirtuin isoforms, three of which are localized to mitochondria (SIRT3, 4, and 5)^4^. Among these, SIRT3 plays a major role in protein deacetylation and the resulting modification of enzymatic activities^5^. The knockout or inhibition of SIRT3 elicits hyperacetylation of mitochondrial proteins, resulting in a decrease in cellular ATP concentration *via* depolarization of the mitochondrial membrane potential^6^,^7^, an increase in the production of reactive oxygen species^8^,^9^, and alteration of mitochondrial morphology^10^,^11^. SIRT3 is thus the main regulator of mitochondrial protein acetylation levels. However, the acetylation processes of mitochondrial proteins remain unclear. While the mitochondrial protein GCN5L1 has been shown to be related to one of the mitochondrial protein acetylation mechanisms^12^, it is also possible that mitochondrial protein acetylation is caused by a reaction between lysine residues and acetyl-CoA in a non-enzymatic process^13^,^14^. Hence, mitochondrial protein acetylation is regulated by both enzymatic and non-enzymatic processes, and controls mitochondrial functions.

“Artificial reaction promoters” are compounds that ptromte chemical reactions *in vitro* and also in cells. In our previous study, we demonstrated that the application of one such artificial reaction promoter, tributylphosphine (PBu_3_), elicited the acetylation of a fluorescent probe in the mitochondria of HeLa cells^15^. PBu_3_ probably enhanced the reactivity of acetyl-CoA in an acetyltransfer reaction in the cells, as well as *in vitro*, and facilitated a chemical reaction between acetyl-CoA and the fluorescent probe in the mitochondria. Based on this result, we hypothesized that PBu_3_ non-enzymatically promotes the acetyltransfer reaction between acetyl-CoA and lysine residues of neighbouring proteins. If PBu_3_ enhances the intracellular non-enzymatic acetyltransfer reaction between those biological molecules, mitochondrial functions might be controlled by the application of an exogenous artificial reaction promoter, since the activities of a number of mitochondrial proteins are regulated by acetylation^16^,^17^,^18^. In this study, we examined the ability of PBu_3_ to promote the acetyltransfer reaction from acetyl-CoA to mitochondrial proteins and to modulate mitochondrial functions in living cells.

## Results

### PBu_3_ promoted protein acetylation in the mitochondria

**Figure 1:**
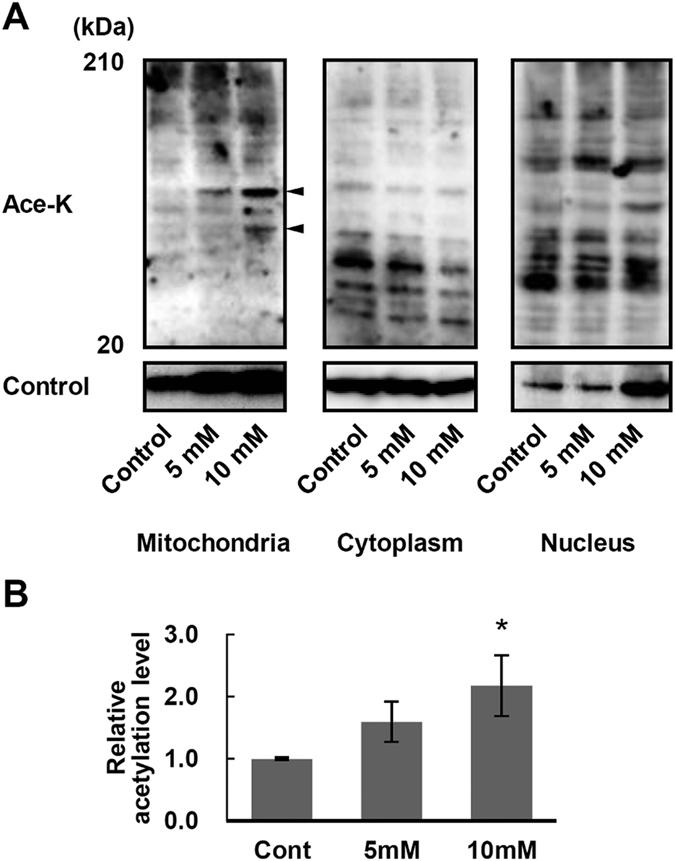
PBu_3_-induced acetylation of mitochondrial proteins. (**A**) Protein acetylation levels were compared using Western blotting probed with anti-acetylated lysine antibody in control and PBu_3_-treated cells (5 mM and 10 mM for 10 min). Mitochondrial, cytoplasmic and nuclear proteins were run under the same experimental conditions in separate runs. Cox4, β-actin and PARP1 were blotted as a loading control for mitochondrial, cytoplasmic and nuclear proteins, respectively. Data are representative of n = 3 experiments. (**B**) Relative acetylation levels in the arrowed bands of mitochondrial protein were calculated from integrated densitometry values relative to Cox4 levels. Data were normalized using control values. PBu_3_ elicited significant protein acetylation in the mitochondria. Bars represent the mean ± SEM of three sets of data run in the same gel. *Indicates *P* < 0.05 (Dunnett’s test).

To verify our hypothesis that PBu_3_ promotes the acetyltransfer reaction between acetyl-CoA and mitochondrial proteins, protein acetylation levels in mitochondria, cytoplasm, and nucleus were estimated by Western blotting using an anti-acetylated lysine antibody. The concentrations of PBu_3_ used were the same as those used in our previous study measuring acetylation reactions using a fluorescent probe^15^. Exposure of HeLa cells to 5 mM and 10 mM PBu_3_ for 10 min elicited acetylation of mitochondrial proteins, especially in the 30–55 kDa range (Fig. 1A). The acetylation signal increased significantly, depending on the PBu_3_ concentration, in the indicated bands of mitochondrial proteins (Fig. 1B). No significant changes in protein acetylation level were observed in the cytoplasm or nucleus (Fig. 1A). Moreover, we observed that mitochondrial superoxide dismutase (SOD2), which is regulated by acetylation^19^, was also acetylated by the 10 min PBu_3_ treatment (see Supplementary Fig. S1), indicating that PBu_3_ induces acetylation of mitochondrial proteins.

**Figure 2:**
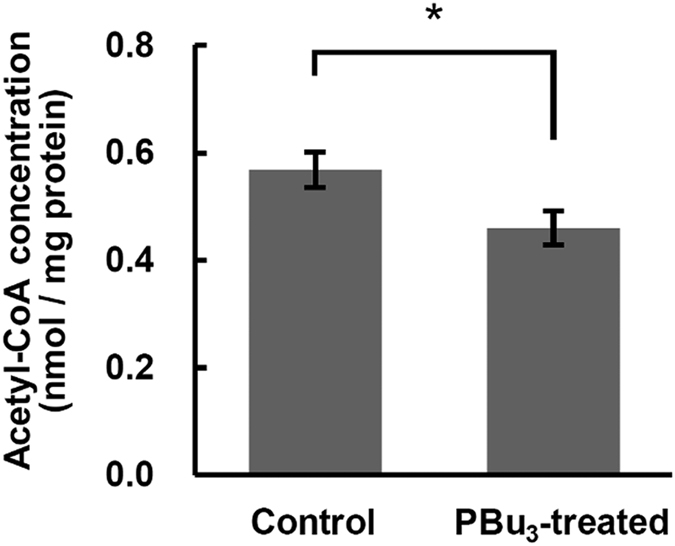
Acetyl-CoA concentration decreased in PBu_3_–treated cells. Cellular acetyl-CoA concentrations were measured and compared between the control and PBu_3_-treated (5 mM for 10 min) cells. Data presented are mean ± SEM of n = 5 samples each. *Indicates *P* < 0.05 (*t*-test).

To confirm that PBu_3_ promotes acetyltransfer reaction between acetyl-CoA and mitochondrial proteins, we estimated cellular acetyl-CoA concentrations (Fig. 2). The concentration decreased in PBu_3_-treated cells, suggesting that acetyl-CoA is the substrate for protein acetylation. However, a high concentration of PBu_3_ might inhibit protein deacetylase, instead of promoting the acetyltranfer reaction, because PBu_3_ also acts as an ion chelator and Zn^2+^ binds to SIRT3^20^. We therefore confirmed that application of PBu_3_ (1–20 mM) has no effect on the activity of SIRT3 *in vitro* (see Supplementary Fig. S2), indicating that PBu_3_ does not inhibit protein deacetylation but promotes protein acetylation. Based on these results, we concluded that PBu_3_ successfully promotes the acetyltransfer reaction from acetyl-CoA to the neighbouring proteins in mitochondria, which probably occurs because of the high concentration of mitochondrial acetyl-CoA.

**Figure 3:**
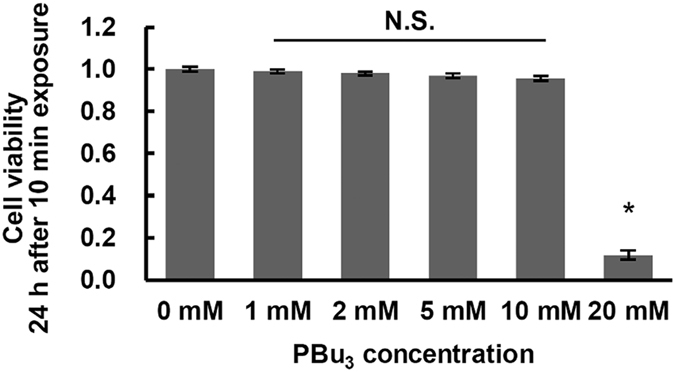
Toxicity of 10 min exposure to PBu_3_. Comparisons of HeLa cell viabilitys 24 h after exposure for 10 min to representative concentrations of PBu_3_, as assessed by MTT assay. Data presented are mean ± SEM of n = 5 samples each from three different experiments. *Indicates *P* < 0.05 (Dunnett’s test).

The toxicity of PBu_3_ was evaluated by exposing HeLa cells to PBu_3_ for 10 min. Concentrations of less than 10 mM had no toxic effect on cell viability after 24 h (Fig. 3). Although exposure to PBu_3_ at concentrations higher than 2 mM for 24 h or 5 mM for longer than 2 h decreased cell viability (see Supplementary Fig. S3), brief treatment to promote protein acetylation in mitochondria (less than 10 mM for 10 min) did not exhibit any toxic effects. These results indicate that short-term exposure to PBu_3_ at an appropriate concentration promotes the acetyltransfer reaction non-invasively in living cells.

### Candidate proteins acetylated by PBu_3_

As shown in Fig. 2A, mitochondrial proteins in the 30–55 kDa range were strongly acetylated by PBu_3_. We therefore aimed to identify the proteins contained in these bands using LC-MS/MS, and succeeded in identifying the following proteins: Succinyl-CoA synthetase subunit β (SUCB1); E3 ubiquitin-protein ligase (MARCH5); monoamine oxidase type A (AOFA); and serine β-lactamase-like protein (LACTB). Among these candidate proteins, SUCB1 is a tricarboxylic acid (TCA) cycle enzyme that catalyses the reaction of succinyl-CoA to succinate, and is involved in ATP production in mitochondria^21^. MARCH5 is involved in mitochondrial quality control and Drp1-dependent mitochondrial fission^22^. These results suggest that PBu_3_-induced acetylation of mitochondrial proteins modifies mitochondrial functions. A number of other proteins are likely to be acetylated by PBu_3_ in addition to the four candidates that we identified

### PBu_3_-induced protein acetylation affected ATP synthesis in mitochondria

**Figure 4:**
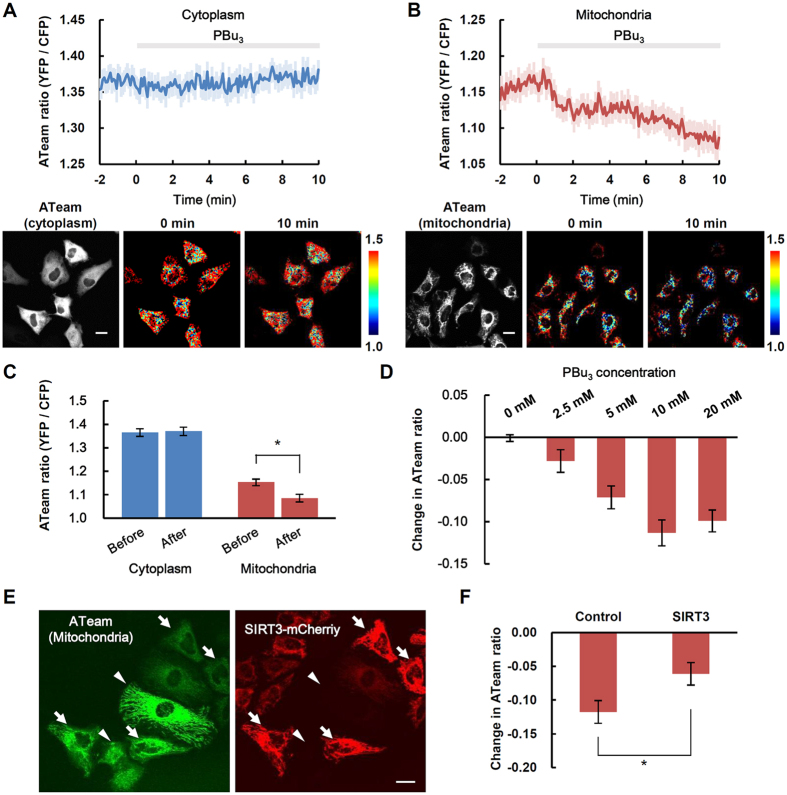
PBu_3_-induced changes in ATP concentrations. (**A**) Time-course of cytoplasmic ATP concentration (mean ± SEM of n = 28 cells from three different experiments) measured using the protein-based ATP sensor ATeam, localized to the cytoplasm (upper). PBu_3_ (5 mM) was applied at 0 min. Representative fluorescence image and pseudo-colored images of an ATeam ratio are shown (lower). Pseudo-colored images show an ATeam ratio (YFP/CFP) ranging from 1.0 (blue) to 1.5 (red) at the indicated time after the application of PBu_3_. (**B**) Time-course of ATP concentration in mitochondria (mean ± SEM of n = 49 cells from five different experiments), measured using ATeam localized to mitochondria. PBu_3_ (5 mM) was applied at 0 min. Representative fluorescence image and pseudo-colored images of an ATeam ratio are shown (lower). (**C**) Comparison of the ATeam ratio (mean ± SEM) before (averaged from −1 to 0 min) and after (averaged from 9 to 10 min) the application of PBu_3_, as shown in A and B. *Indicates *P* < 0.05 in *t*-test. (**D**) Change in the mitochondria-localized ATeam ratio (mean ± SEM) after 10 min in response to the indicated concentrations of PBu_3_ (0 mM: n = 78 cells from nine different experiments; 2.5 mM: n = 21 cells from four different experiments; 5 mM: n = 42 cells from four different experiments; 10 mM: n = 37 cells from four different experiments; and 20 mM: n = 23 cells from two different experiments). (**E**) ATeam (left) and SIRT3-mCherry (right) fluorescence images of the same region. ATeam signal was compared between SIRT3-mCherry overexpressing cells (arrows) and non-overexpressing cells (arrowheads). (**F**) Comparison of ATeam ratio (mean ± SEM) before (averaged from −1 to 0 min) and after (averaged from 9 to 10 min) application of PBu_3_ (n = 27 cells for Control and 26 cell for SIRT3 from nine different experiments). *Indicates *P* < 0.05 (*t*-test). Scale bar indicates 20 μm.

It has been reported that the cellular ATP concentration is lower in SIRT3 knockout cells than that in normal cells^10^, indicating that protein acetylation inhibits ATP synthesis in mitochondria. We therefore examined the effect of PBu_3_-induced protein acetylation on ATP concentration using the ATP sensor protein, ATeam^23^. While 5 mM PBu_3_ had no effect on the ATP concentration in cytoplasm (Fig. 4A), it elicited a gradual but significant decrease in the ATP concentration in mitochondria (Fig. 4B,C). The mitochondrial ATP concentration was lower than that in cytoplasm as reported before^23^ (Fig. 4C). PBu_3_ decreased the mitochondrial ATP concentration in a dose-dependent manner (0–10 mM; Fig. 4D).

To ascertain whether the PBu_3_-induced decrease in ATP concentration was the caused by protein acetylation in the mitochondria, we compared changes in ATP concentration resulting from PBu_3_ application between normal cells and SIRT3-overexpressing cells (Fig. 4E). The decrease in ATP concentration was partially suppressed in the SIRT3-overexpressing cells, since it was attenuated by the protein deacetylase SIRT3 (Fig. 4F), indicating that PBu_3_ modulates ATP concentration *via* mitochondrial protein acetylation.

The protein acetylation induced by PBu_3_ thus resulted in a decrease in mitochondrial ATP concentration, probably due to the inhibition of enzymes involved in ATP production by acetylation: ATP synthase; the enzymes in the TCA cycle; and the electron transport chain^24^,^25^,^26^,^27^. Pharmacological inhibition of mitochondrial ATP synthesis by oligomycin induced a similar magnitude of decrease in ATP concentration, and the collapse of the mitochondrial inner membrane potential induced by carbonyl cyanide p-(trifluoromethoxy) phenylhydrazone (FCCP) elicited a greater decrease in ATP (see Supplementary Fig. S4). While the levels of decrease were comparable to those induced by PBu_3_, the rate of decrease induced by these inhibitors was faster, suggesting that the inhibition of mitochondrial ATP synthesis by PBu_3_ is moderate by comparison. Moreover, we observed PBu_3_-induced modulation of mitochondrial ATP concentration in cells of the non-cancerous tissue-derived cell line HEK293 (see Supplementary Fig. S5).

**Figure 5:**
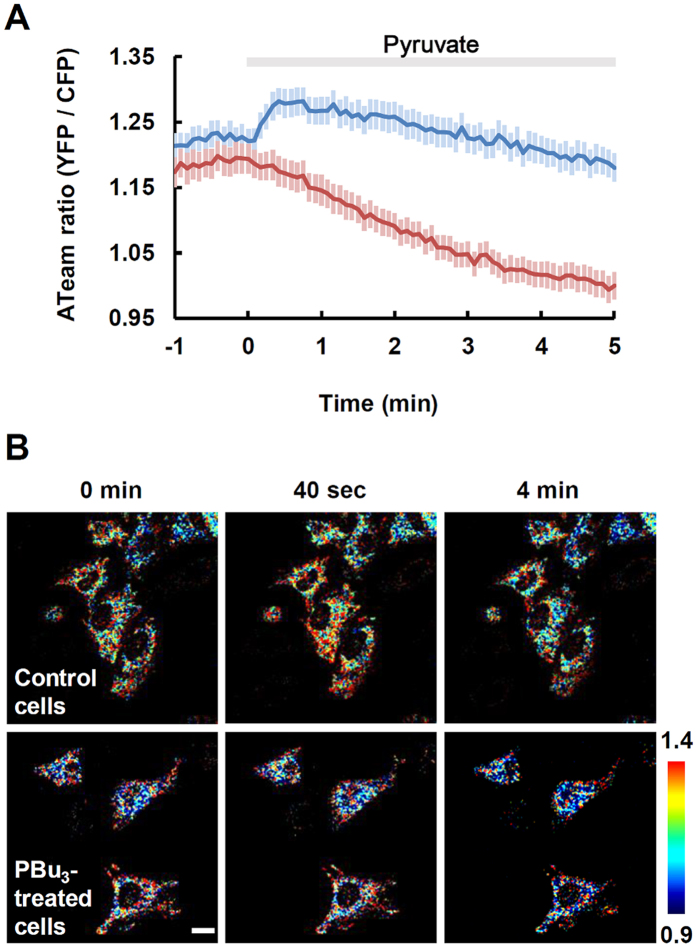
Pre-treatment with PBu_3_ changed the response to pyruvate. (**A**) Time-course of the change in mitochondrial ATP concentration (mean ± SEM) in control cells (blue line; n = 61 cells from five different experiments) and PBu_3_-treated cells (red line; n = 51 cells from five different experiments). The PBu_3_-treated cells were exposed to 5 mM of PBu_3_ 10 min prior to the fluorescence measurements. Pyruvate (5 mM) was applied at 0 min. (**B**) Pseudo-colored images of control cells (upper panel) and PBu_3_-treated cells (lower panel). Pseudo-colored images show an ATeam ratio (YFP/CFP) ranging from 0.9 (blue) to 1.4 (red) at the indicated time after the application of pyruvate (5mM). Scale bar indicates 20 μm.

PBu_3_ therefore promoted the acetyltransfer reaction from acetyl-CoA to mitochondrial proteins, which inhibited ATP production in mitochondria. Although acetyl-CoA is normally an essential substrate in mitochondrial energy production, mitochondrial ATP concentrations are decreased by PBu_3_-induced protein acetylation involving acetyl-CoA. We next assessed whether the dominant role of acetyl-CoA in the PBu_3_-treated cells was to act as a substrate for the TCA cycle or protein acetylation. To address this, changes in the mitochondrial ATP concentration in response to pyruvate were compared between control and PBu_3_-treated cells, because acetyl-CoA is produced through pyruvate decarboxylation. In the control cells, pyruvate (5 mM) induced a transient increase in mitochondrial ATP concentration (Fig. 5A blue line and B upper panels). In contrast, it decreased ATP concentrations in the PBu_3_-treated cells (Fig. 5A red line and B lower panels). These results indicate that acetyl-CoA contributes predominantly to protein acetylation in the presence of PBu_3_, which results in the further decrease in ATP concentrations.

### PBu_3_-induced protein acetylation elicited alterations in mitochondrial morphology

**Figure 6:**
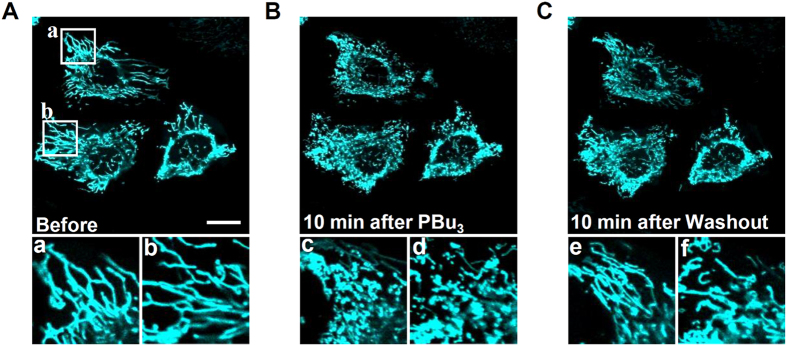
PBu_3_ induced reversible mitochondrial fragmentation. The mitochondrial shape was visualized using TagCFP-mito. (**A**) Elongated mitochondria were observed prior to the application of PBu_3_. (**B**) Fragmented mitochondria were observed after incubation in PBu_3_ (5 mM) containing medium for 10 min at 37 °C on a stage top incubator. (**C**) 10 min after the PBu_3_ was washed out, the mitochondria returned to a linear shape. (a–f) Magnified areas of the images (a,b in **A**; c,d in **B**; and e,f in **C**). Scale bar indicates 20 μm. Data in this figure are representative of five experiments.

Recent studies have reported that the acetylation and deacetylation of mitochondrial proteins regulates mitochondrial fusion and fission, resulting in alterations in mitochondrial morphology^10^,^11^. These studies showed that mitochondria are fragmented in cells defective for SIRT3 or with a mutation in its downstream protein, and indicated that hyperacetylation elicits mitochondrial fragmentation, while deacetylation reverses this process. To demonstrate this process directly, we monitored mitochondrial shapes with mitochondria targeted TagCFP, before and after the application of PBu_3_. PBu_3_ induced mitochondrial fragmentation within 10 min (Fig. 6A,B). Furthermore, the mitochondria returned to their normal shapes in 10 min after the PBu_3_ was washed out (Fig. 6C). In SIRT3-overexpressing cells, the effect of PBu_3_ appeared to be attenuated (see Supplementary Fig. S6). These results indicate that PBu_3_-induced protein acetylation reversibly regulates mitochondrial morphology, and that acetylation-induced mitochondrial morphological change are occurs quickly, within 10 min. Based on these results, we conclude that our method involving PBu_3_ successfully modulates mitochondrial functions *via* mitochondrial protein acetylation, which enabled us to observe the time-course of the effects in mitochondria.

## Discussion

**Figure 7:**
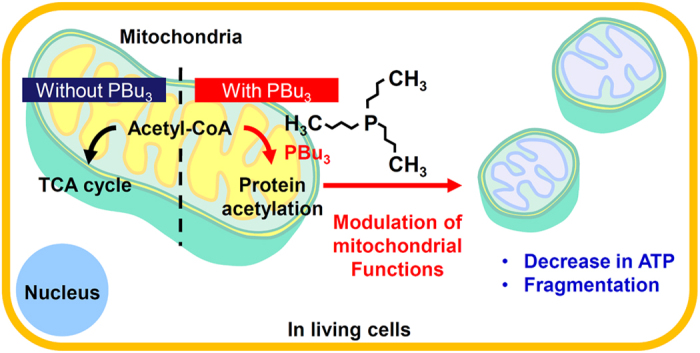
Schematic diagram of the results of this work.

In this study, we have shown that PBu_3_ promotes mitochondrial protein acetylation and modulates mitochondrial functions. PBu_3_ has been used as a catalyst in the acylation reaction^28^. It also catalyses the reaction in living cells, as shown in our previous study using a newly developed fluorescent probe^15^. The chemicals that promote the specific reaction intracellularly are referred to as “artificial reaction promoters”. In our previous studies, these molecules were used to promote the reaction between specific biological molecules and fluorescent probes, which allowed us to measure biological molecules, such as acetyl-CoA and NAD(P)H, using the fluorescence imaging method^15^,^29^. In this study, the artificial reaction promoter, PBu_3_, was used to non-invasively promote the reaction between the biological molecules, acetyl-CoA and mitochondrial proteins (Figs 1, 2, 3), and to modulate mitochondrial functions in living cells (Figs 4, 5, 6) as summarized in Fig. 7. Our data show that PBu_3_ promotes the reaction between acetyl-CoA and mitochondrial proteins, and modulates mitochondrial functions, at least in part, *via* the protein acetylation, although there might be other route. PBu_3_ is therefore a useful tool for the control of cellular functions. To our knowledge, this is the first report to demonstrate the modulation of cellular function *via* the promotion of a specific chemical reaction in living cells.

While the protein acetylation process in mitochondria is not fully understood, the non-enzymatic chemical reaction between acetyl-CoA and lysine residues might be sufficient to explain mitochondrial protein acetylation^30^. If there is a sufficient amount of acetyl-CoA to maintain the protein acetylation state in mitochondria in contrast to the other compartments in the cell, it makes sense that PBu_3_ induces protein acetylation specifically in mitochondria. Acetylation of mitochondrial proteins negatively regulates mitochondrial functions in many cases^14^. In this study, we demonstrated that PBu_3_-induced acetylation down-regulates mitochondrial ATP production (Figs 4 and 5) and elicits mitochondrial fragmentation (Fig. 6) in HeLa cells. Although energy metabolism in cancer cell lines is different from that in non-cancerous cells, we observed this effect of PBu_3_ on mitochondrial ATP concentration in both the cancerous HeLa cells and the non-cancerous HEK293 cells (see Supplementary Fig. S5). These results indicate that the effects of PBu_3_ shown here are not unique to cancer cells. In addition to the processes observed in this study, mitochondrial protein acetylation is also related to oxidative damage^11^,^31^, fatty acid oxidation^2^, mitochondrial autophagy^32^,^33^, and apoptosis^17^. These mechanisms are important for maintaining the normal functions of cells and tissues; hence, abnormal acetylation of mitochondrial proteins has been implicated in a number of diseases, such as metabolic syndromes^34^, diabetes^35^, Parkinson’s disease^36^, and Alzheimer’s disease^37^. Using PBu_3_ at suitable concentrations, protein acetylation levels in mitochondria can be reversibly controlled without the knockout or inhibition of acetyltransferase. Reversible regulation of this significant physiological process might therefore be a powerful tool for investigating the pathogenesis of these diseases.

Recent studies have reported methods referred to as “bioorthogonal chemistry”, which allow artificial reactions to proceed in the cellular environments^38^,^39^. These methods enable the occurrence of specific reactions between artificially-induced compounds, or between endogenous molecules and artificially-induced compounds, for tagging and probing intracellular molecules in living cells^40^,^41^. In contrast, our method enhances a specific reaction between intrinsic biological molecules in living cells. Modulating biological reactions and functions without the knockout or inhibition of proteins is a novel approach to understanding intracellular events and physiological functions in living cells. We refer to this concept as “bioparallel chemistry”^29^. With the use of artificial reaction promoters, cellular functions other than protein acetylation might be controlled. These methods can reveal novel aspects of chemical reactions under physiological conditions, and allow for the control of cellular functions.

## Methods

### Cell Culture

HeLa cells and HEK293 cells were cultured in DMEM supplemented with 10% (v/v) FBS and 1% (v/v) penicillin/ streptomycin in an incubator maintained at 37 °C and with a humidified atmosphere of 5% CO_2_. For the fluorescence measurements, the cells were seeded onto glass-based dishes.

### Fluorescence measurements

Changes in cytoplasmic and mitochondrial ATP concentrations were measured using an ATP sensor protein, ATeam, localized to the cytoplasm and mitochondria, respectively^23^. Mitochondrial shapes in the HeLa cells were visualized using TagCFP-mito (Evrogen, Moscow, Russia). The plasmids coding for ATeam or TagCFP-mito were transfected into HeLa cells using Lipofectamine LTX (Invitrogen, Carlsbad, CA, USA) one day before the fluorescence measurements were conducted. DNA coding human SIRT3 was cloned from HeLa cell cDNA and inserted to the pmCherry-N1 vector using the BglII and EcoRI restriction enzyme sites, then transfected into HeLa cells following ATeam-transfection. The bath solution was changed to Hanks’ balanced salt solutions (HBSS) containing (in mM): NaCl, 137; KCl, 5.4; CaCl_2_, 1.3; MgCl_2_, 0.5; MgSO_4_, 0.4; Na_2_HPO_4_, 0.3; KH_2_PO_4_, 0.4; NaHCO_3_, 4.2; D-Glucose, 5.6; HEPES, 5 (pH adjusted to 7.4 with NaOH) before the fluorescence measurements were conducted.

Fluorescence imaging was performed using a confocal laser scanning microscope system (FluoView FV1000; Olympus, Tokyo, Japan) mounted on an inverted microscope (IX81; Olympus) with 40 × and 60 × oil–immersion objective lenses. The temperature of the microscope stage was maintained at 37 °C during the experiments using a stage top incubator (IN-OIN-F2, Tokai hit, Shizuoka, Japan). TagCFP-mito was excited at 440 nm with a laser diode, and a signal was observed at 460–560 nm. ATeam was excited at 440 nm, and the fluorescence signals were separated using a 510 nm dichroic mirror and observed at 460–500 nm for CFP and 515–615 nm for YFP. Fluorescence images were acquired and analysed with the FluoView software package (Olympus). Fluorescence intensities were calculated as mean intensity over a defined region of interest (ROI) containing the entire cell body of each cell.

### Western Blotting

Control and PBu_3_-treated cells were harvested and the mitochondria, cytoplasm, and nucleus isolated using the Mitochondria Isolation Kit (BioChain Institute, Gibbstown, NJ, USA). The samples were lysed in RIPA buffer containing 25 mM HEPES, 1.5% TritonX-100 (v/v), 1.0% sodium-deoxycholate (w/v), 0.1% SDS (w/v), 500 mM NaCl, 5 mM EDTA, 50 mM NaF, 100 μM Na_3_VO_4_, and 0.1 mg/mL leupeptin and protease inhibitor cocktail (Nacalai Tesque, Kyoto, Japan). The protein lysates were diluted to the same protein concentrations, separated using SDS-PAGE, transferred onto a PVDF membrane (Millipore, Billerica, MA, USA), and probed with an acetylated lysine-specific antibody (Sigma-Aldrich, St. Louis, MO, USA). The secondary antibody used was a horseradish peroxidase (HRP)-conjugated anti mouse IgG (GE Healthcare, Little Chalfont, UK). The ECL Western blotting detection system (Millipore) was used for detection with imaging by LAS-1000 (Fuji Film, Tokyo, Japan). After detecting acetylated lysine signals, the HRP conjugated to the secondary antibody was inactivated by incubating the membrane in 15% H_2_O_2_ for 30 min. Loading control proteins were then probed with a β-actin-specific antibody for the cytoplasmic protein sample, a Cox4-specific antibody for the mitochondrial protein sample, and a PARP1-specific antibody for the nuclear protein sample (GeneTex, Irvine, CA, USA). The secondary antibody was HRP-conjugated anti rabbit IgG (GE Healthcare) and the signals were detected as described above.

### Quantification of Acetyl-CoA

Control and PBu_3_-treated (5 mM for 10 min) cells were harvested in ice-cold PBS and sonicated. The protein concentration of each sample was estimated using Coomassie Brilliant Blue (CBB) protein assay. The cell lysates were deproteinised using the Deproteinising Sample Preparation kit (BioVision, Milpitas, CA, USA). The acetyl-CoA concentration was then quantified using the PicoProbe Acetyl CoA Assay kit (BioVision). The probe was excited at 535 nm and the fluorescence measured at 589 nm using a microplate reader (Fluoroskan Ascent FL, Thermo Fisher Scientific, Waltham, MA, USA). The concentration was normalized using the protein concentration of each sample.

### Identification of acetylated proteins

Mitochondrial protein samples were separated using SDS-PAGE, and the gel was stained using the Silver Stain MS kit (Wako, Osaka, Japan). The gel was cut at an appropriate position, and proteins contained in the gel fragment were digested using 20 ng/mL Trypsine. The digested proteins were eluted and resolved in elution buffer (50% acetonitrile and 5% trifluoroacetic acid). The sample was analysed using LC-MS/MS (Impact HD, Bruker Daltonics, Billerica, MA, USA).

### Measurement of cell viability

Cell viability was measured using the MTT assay. After the cells had been exposed to PBu_3_-containing medium, this was replaced with a medium containing 0.5 mg/mL of MTT. The cells were incubated for 2 h at 37 °C. The medium was removed and 100 μL of DMSO was added to each well to dissolve the precipitate. Absorbance at 570 nm was measured using a microplate reader. Values from control cells were used to estimate the cell viability.

### Statistical Analysis

Significant differences between two data-sets were determined using the Student’s *t*-test, and the Dunnett’s test was used for multiple comparisons. *P* values lower than 0.05 were considered significant.

## Additional Information

**How to cite this article**: Shindo, Y. *et al*. An Artificial Reaction Promoter Modulates Mitochondrial Functions via Chemically Promoting Protein Acetylation. *Sci. Rep.* **6**, 29224; doi: 10.1038/srep29224 (2016).

## Supplementary Material

Supplementary Information
