# Supplementary material for: An Artificial Reaction Promoter Modulates Mitochondrial Functions via Chemically Promoting Protein Acetylation
Source: Sci Rep. 2016 Jul 4;6:29224. doi: 10.1038/srep29224 (PMC4931687; doi:10.1038/srep29224)
Supplement: Supplementary Information [file srep29224-s1.pdf]

Supporting Information

An Artificial Reaction Promoter Modulates Mitochondrial Functions via Chemically Promoting Protein Acetylation

Yutaka Shindo, Hirokazu Komatsu, Kohji Hotta, Katsuhiko Ariga, Kotaro Oka*

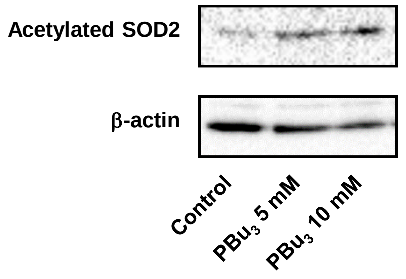

Figure S1 PBu_3-induced acetylation of SOD2

Acetylation level of mitochondrial superoxide dismutase, SOD2, was compared using Western blotting probed with anti-acetylated SOD2 antibody (Abcam, Cambridge, UK) in the control and PBu_3-treated cells (5 mM and 10 mM for 10 min). β-actin was blotted as a loading control. Acetylation level of SOD2 was increased depending on the concentration of PBu_3.

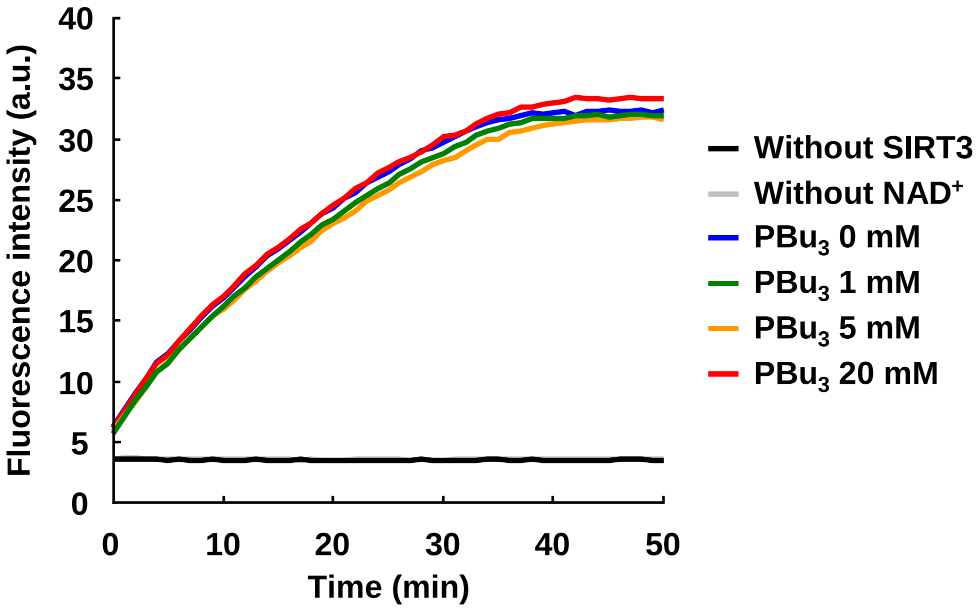

Figure S2 PBu_3 had no effect on the activity of protein deacetylase, SIRT3.

Activity of SIRT3 was measured using the SIRT3 deacetylase fluorometric assay kit (CycLex, Nagano, Japan). The activity of SIRT3 at various PBu_3 concentrations was measured as fluorescence intensity using a microplate reader (Fluoroskan Ascent FL, Thermo Fisher Scientific, Waltham, MA, USA). SIRT3 was added to each sample immediately before measurements were conducted. The probe was excited at 355 nm and fluorescence at 450 nm was measured every minute. PBu_3 in a range from 0–20 mM had no effect on the activity of SIRT3.

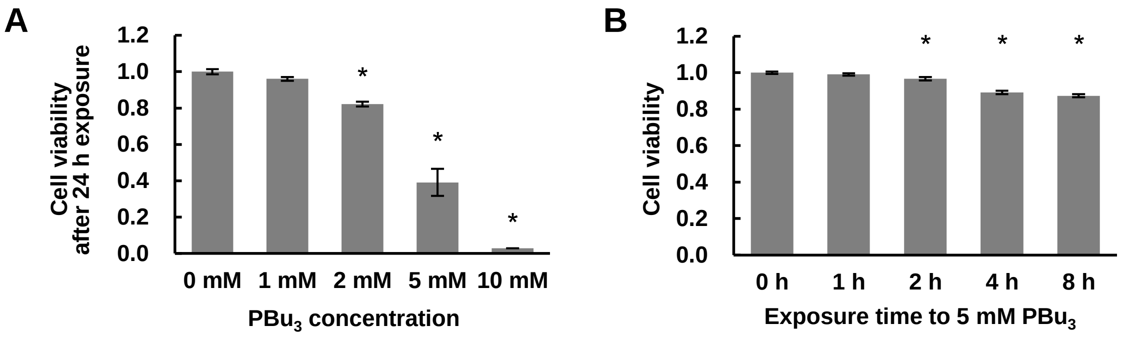

Figure S3 Toxicity of PBu_3 measured by MTT assay.

(A) Cell viabilities after 24 h exposure to indicated concentration of PBu_3 containing medium. Data are represented as mean ± SEM of n = 10 samples from three different experiments for 0–5 mM and n = 6 samples from two different experiments for 10 mM. (B) Viabilities of HeLa cells exposed to 5 mM of PBu_3 for indicated time. Data are represented as mean ± SEM of n = 16 samples from four different experiments. * indicates $P < 0.05$ (Dunnett's test).

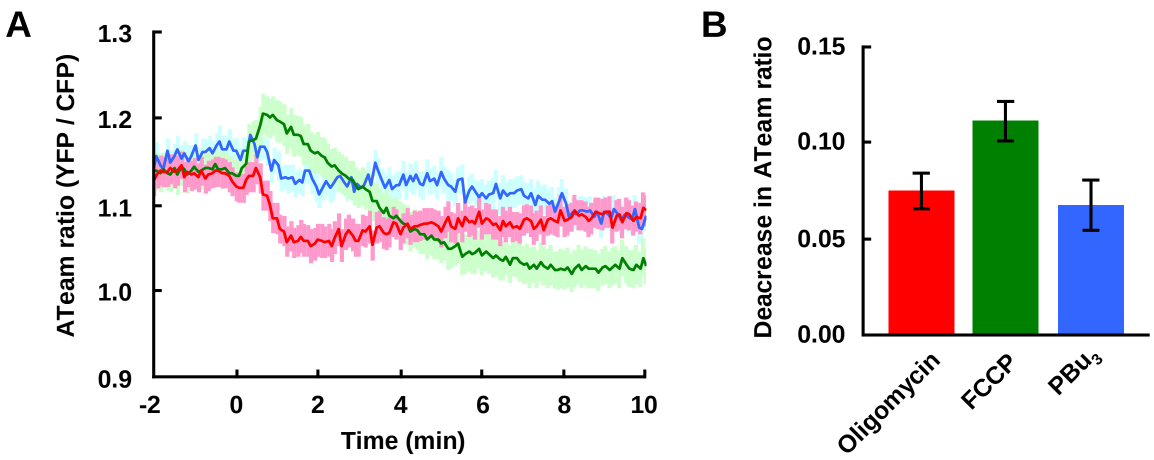

Figure S4 Changes in mitochondrial ATP concentration induced by several inhibitors of mitochondrial function.

(A) Time-courses of the change in mitochondrial ATP concentration in response to oligocymin, which is an inhibitor of ATP synthase on mitochondrial inner membrane (10 μM, red line), carbonyl cyanide p-(trifluoromethoxy) phenylhydrazone (FCCP), which is an uncoupler of mitochondrial membrane potential (5 μM, green line), and PBu_3 (5 mM, blue line). (B) Comparison of the decrease in ATeam ratio. Difference in the ATeam ratio between the average value from -1 to 0 min and that from 1 to 2 min was represented for oligomycin. Differences in the ATeam ratio between the average value from -1 to 0 min and that from 9 to 10 min were represented for FCCP and PBu_3. Data are represented as mean ± SEM of n = 55 cells from five experiments for oligomycin, n = 49 cells from five experiments for FCCP, and n = 49 cells from five experiments for PBu_3. The data for PBu_3 is the same data shown in Figure 5B in main manuscript.

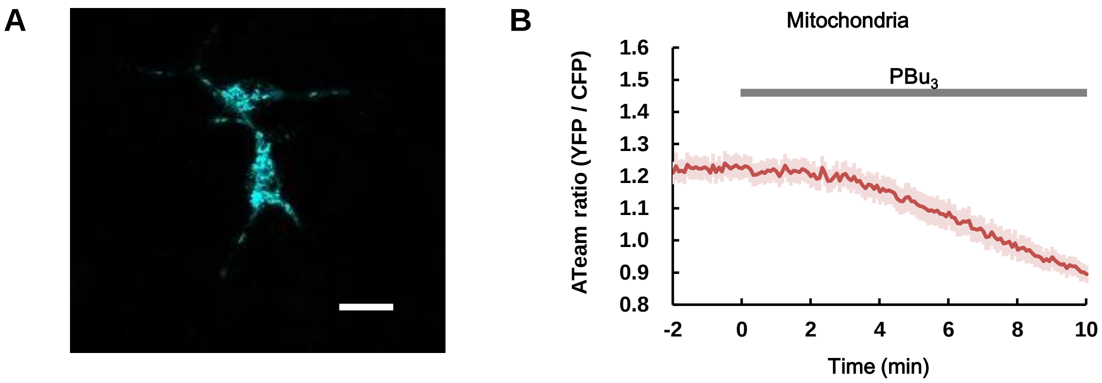

Figure S5 PBu$_3$ induced decrease in mitochondrial ATP concentration in non-cancerous tissue-derived cell line, HEK293 cells.

(A) Fluorescence image of mitochondria targeted ATeam-expressing HEK293 cells. Scale bar indicates 20 μm. (B) Time-course of ATeam ratio (ATP concentration in mitochondria) in response to PBu$_3$ (5mM). Data are represented as mean ± SEM of n = 21 cells from four different experiments.

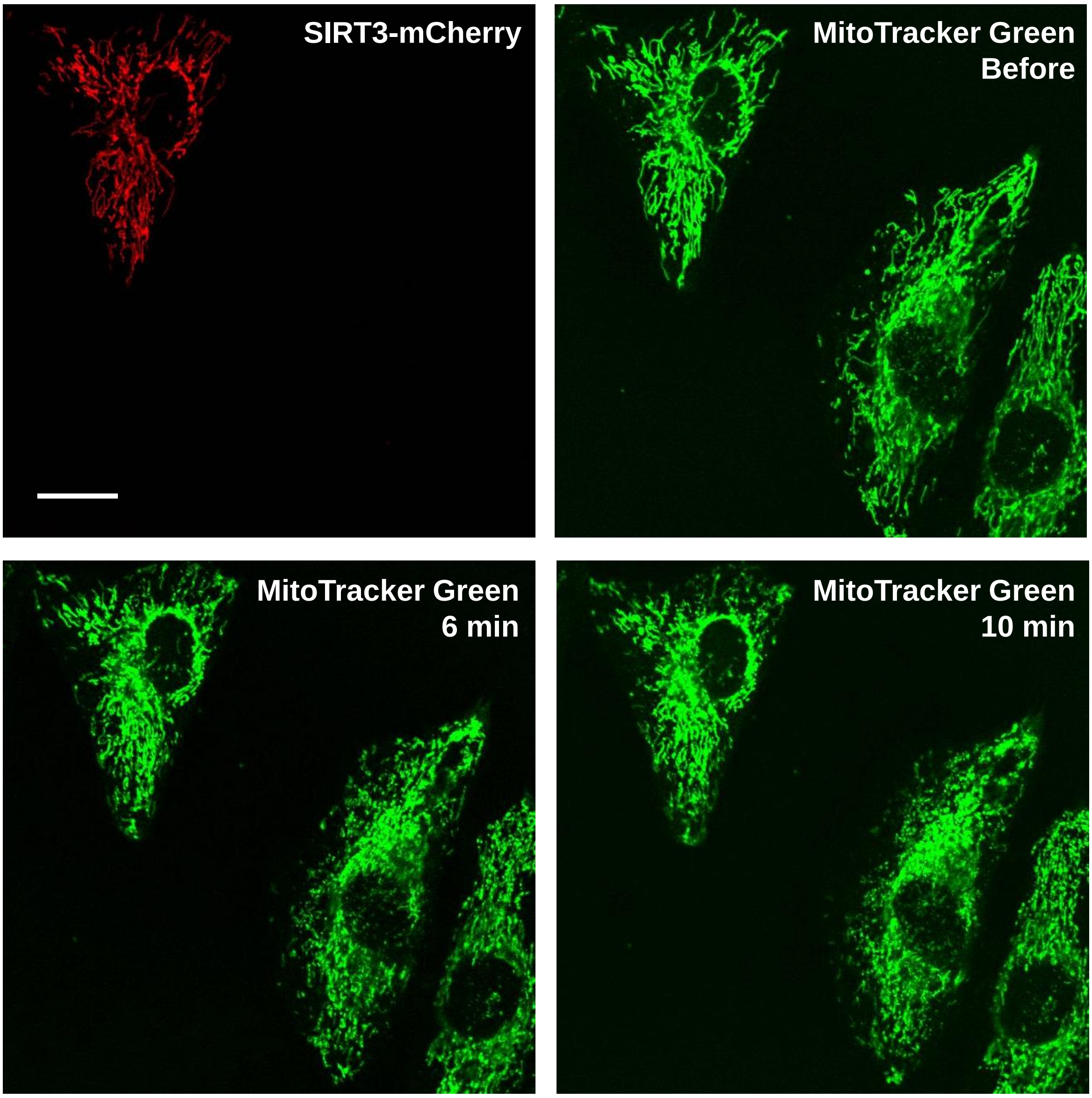

Figure S6 Comparison of PBu_3-induced mitochondrial morphological change in normal and SIRT3 overexpressing HeLa cells.

Fluorescence image of SIRT3-mCherry (upper left) indicates one cell in this viewing field overexpress SIRT3. Mitochondria were labeled with MitoTracker Green FM and observed before and after (6 and 10 min) PBu_3 treatment. While SIRT3 overexpressing cell maintained mitochondrial shape, mitochondria in non-overexpressing cells fragmented. Data in this figure are representative of three experiments. Scale bar indicates 20 μm.
